# Scion, Rootstock and Their Interaction Affect the Photosynthesis of Citrus

**DOI:** 10.3390/plants14172718

**Published:** 2025-09-01

**Authors:** Shiping Zhu, Mengyu Liu, Guotao Luo, Zhou Hu, Xiaonan Zhang, Jinsong Xiang, Rong Yang, Shixue Hu, Xiaodong Cai, Xin Yu

**Affiliations:** 1 Citrus Research Institute, Southwest University/Chinese Academy of Agricultural Sciences, Beibei, Chongqing 400712, China; 2 National Citrus Engineering Research Center, Beibei, Chongqing 400712, China; 3 Fengjie Citrus Research Institute, Fengjie, Chongqing 404600, China; 4 College of Horticulture and Gardening, Yangtze University, Jingzhou 434025, Hubei, China

**Keywords:** scion–rootstock interaction, photosynthesis, grafting, field conditions, electron transport rate

## Abstract

Photosynthesis is an essential plant biological process. The performance of photosynthesis in grafted plants is affected by either the scion or the rootstock. However, the effect of the scion, rootstock and their interaction in the scion–rootstock combinations on photosynthesis of the grafted trees was not clear. In this research, the photosynthesis was analyzed within 21 citrus scion–rootstock combinations derived from three navel oranges (*Citrus sinensis* cv. ‘Banfield’, ‘Chislett’ and ‘Powell’) grafted on seven rootstocks [(Swingle citrumelo (*C*. *paradisi* × *Poncirus trifoliata*), Carrizo citrange (*C*. *sinensis* × *P*. *trifoliata*), X639 (*C*. *reticulata* × *P*. *trifoliata*), MXT (*C*. *sinensis* × *P*. *trifoliata*), Hongju (*C*. *reticulata*), Ziyang xiangcheng (*C*. *junos*) and Trifoliate orange (*P*. *trifoliata*)]. Results indicated that photosynthesis of these grafted citrus plants was significantly affected by all the scion, rootstock and their interaction. The rootstock and scion–rootstock interaction had more effect on both chlorophyll fluorescence and photosynthetic parameters with lower p values than the scion. All the scions grafted on Swingle showed the highest electron transport rate at 132.24, 158.39 and 154.59 µmol electrons m^−2^ s^−1^, and a higher net CO_2_ assimilation rate at 11.22, 10.77 and 11.69 µmol m^−2^ s^−1^, respectively. The rootstock is the predominant factor affecting the content of photosynthetic pigments, and the combinations using Ziyang xiangcheng as the rootstock had the highest content at 19.83, 20.97 and 20.39 μmol s^−1^ Kg^−1^ FW. Electron transport rate is probably the predominant factor determining the final photosynthesis of the grafted citrus trees. This research is the first to reveal the respective effect of the scion, rootstock and their interaction on photosynthesis of citrus scion–rootstock combinations and is valuable in enhancing the understanding of the different performances in citrus scion–rootstock combinations, which aids in selecting optimal scion–rootstock combinations.

## 1. Introduction

Photosynthesis is a vital biological process for plants to convert light energy into organic matter, which is essential for plant growth [1,2,3]. It begins with light absorption of pigments in photosystems and then continues with the two stages of light reaction and dark reaction, which react on thylakoid membranes and chloroplast stroma, respectively. During the first stage, the light quantum absorbed by photosynthetic pigments experiences four pathways: heat dissipation, fluorescence loss, exciton transferring and electron transportation. The second stage is the process of CO_2_ assimilation via Calvin cycle, in which the enzyme Rubisco is involved [4].

Photosynthetic pigments such as chlorophyll and carotenoids play indispensable roles in absorbing light quanta, transferring energy, phosphorylation and CO_2_ assimilation [5,6,7]. Non-photochemical quenching (NPQ) is an important process for plants to dissipate and release the excess photons absorbed by Chl antenna [8]. The parameter of fluorescent quenching (qP) reflects the light energy, which dissipates as heat or electron transport [9]. The maximal quantum yield of PSII photochemistry (Fv/Fm) shows the activity of the PSII components and indicates whether the plant is under stress [10]. Leaf photosynthesis is subject to both stomatal and non-stomatal limitations [11]. The former refers to the restrictions in CO_2_ supply by diffusion via the stomata to the intercellular spaces of leaves, while the latter is associated with the diffusion of CO_2_ from the leaf-intercellular spaces to the sites of the dark reactions of photosynthesis in chloroplasts, and biochemical limitations of photosynthetic efficiency [12]. Therefore, many factors such as temperature, light intensity, CO_2_ concentration, activity of photosynthetic enzymes and pigment content can affect plant photosynthesis. Grafting is an ancient technique that joins two plants into a new one [13]. Nowadays, most fruit trees are propagated by this technique. The growth, fruit quality [14,15,16,17] and stress tolerance [18,19,20,21] of the grafted plants are affected by both the rootstock and the scion or by them individually [22,23,24]. The interaction of rootstock and scion could affect the efficiency of the photosynthesis [25,26,27,28]. Therefore, selecting the optimal scion–rootstock combination could improve the plant growth, fruit yield and quality and stress tolerance by the enhancement of photosynthesis.

Citrus, the most important fruit crop worldwide, is also propagated by grafting. The influences of citrus rootstocks on tree size, fruit yield and quality [17,29,30,31,32,33] and stress tolerance [21,31,34,35,36] of scion cultivars have been extensively studied. However, the research on the effect of scion–rootstock interaction on photosynthesis under natural field conditions has rarely been conducted.

The present study investigated the photosynthesis of 21 different citrus scion–rootstock combinations to analyze the effect of scion, rootstock and their interaction on the performance of photosynthesis, aiming at better understanding the performance difference of different scion–rootstock combinations in physiology and providing a reference for selecting optimal scion–rootstock combinations in citriculture.

## 2. Results

### 2.1. Photosynthetic Parameters of the Citrus Scion–Rootstock Combinations

Scion, rootstock and their interaction significantly affected the photosynthetic parameters (Table 1). Rootstock had the greatest effect with the lowest p values on the rate of net CO_2_ assimilation and the stomatal conductance, while the interaction of scion and rootstock demonstrated the greatest impact on the concentration of intercellular CO_2_ and the rate of transpiration. However, scion showed the lowest but significant effect on A_CO2_, G_H2O_ and E but was not significant on Ci. The results indicated that the effect of both rootstock and scion–rootstock interaction was greater than that of scion on the parameters of photosynthesis. The photosynthetic parameters varied among the 21 scion–rootstock combinations. The highest rate of net CO_2_ assimilation was observed in Swingle combined with both Banfield and Chislett (BF/SW, CH/SW) at 11.22 and 10.77 µmol m^−2^ s^−1^, respectively, and MXT with Powell (PW/MXT) at 12.27 µmol m^−2^ s^−1^. Hongju, Trifoliate orange and Ziyang xiangcheng combined with the three scions (BF/HJ, CH/TO and PW/ZY) had the lowest A_CO2_ at 6.67, 5.58 and 7.07 µmol m^−2^ s^−1^, respectively. The highest stomatal conductance was also found in Banfield and Chislett grafted on Swingle (BF/SW, CH/SW) at 163.92 and 159.31 mmol m^−2^ s^−1^, respectively, and Powell on Trifoliate orange (PW/TO) at 153.97 mmol m^−2^ s^−1^. The lowest G_H2O_ values of the scions were shown in Chislett and Powell grafted on Ziyang xiangcheng (CH/ZY, PW/ZY) at 90.34 and 97.38 mmol m^−2^ s^−1^, respectively, and Banfield on Hongju (BF/HJ) at 96.73 mmol m^−2^ s^−1^, which were significantly lower than those of the three scions grafted on the other rootstocks. The rootstock X639 grafted with both Banfield and Powell (BF/X639, PW/X639) had the highest concentration of intercellular CO_2_ at 353.97 and 355.35 μmol mol^−1^, respectively, while Trifoliate orange with Chislett (CH/TO) had the highest Ci at 388.59 μmol mol^−1^. The combinations of BF/TO, CH/HJ and PW/CA had the lowest Ci at 310.34, 304.88 and 304.63 μmol mol^−1^, respectively. The highest transpiration rate was detected in the combinations of BF/SW, CH/639, PW/MXT at 4.30, 3.16 and 3.91 mmol m^−2^ s^−1^, respectively, and the lowest E values were obtained in BF/HJ, CH/MXT and PW/ZY at 1.74, 1.68 and 1.78 mmol m^−2^ s^−1^.

### 2.2. Chlorophyll Fluorescence of the Citrus Scion–Rootstock Combinations

Scion, rootstock and scion–rootstock interaction all had significant effects on electron transport rate; however, rootstocks made the greatest contributions to ETR than the other two factors with the lowest p values. Among the seven rootstock varieties, Swingle possessed the highest rate of electron transport with all the three scions (BF/SW, CH/SW, PW/SW) at 132.24, 158.39 and 154.59 µmol electrons m^−2^ s^−1^, respectively. Ziyang xiangcheng combined with Chislett and Powell (CH/ZY, PW/ZY), and Hongju with Banfield (BF/HJ) had the lowest ETR at 90.22, 83.00 and 81.88 µmol electrons m^−2^ s^−1^, respectively, which were significantly lower than that of the other rootstocks combined with the three scions. Only rootstock had a significant effect on the quantum yield of the PSII. Swingle grafted with the three scions (BF/SW, CH/SW, PW/SW) also possessed the highest quantum yield of the PSII at 0.32, 0.38 and 0.38, respectively, while Ziyang xiangcheng with Chislett and Powell (CH/ZY, PW/ZY) and Hongju with Banfield (BF/HJ) had the lowest values at 0.22, 0.20 and 0.20, respectively. Both scion and scion–rootstock interaction had a significant effect on the maximal quantum yield of PSII photochemistry and non-photochemical quenching of fluorescence. Trifoliate orange grafted with Banfield (BF/TO) and Carrizo with both Chislett and Powell (CH/CA, PW/CA) showed significantly lower Fv/Fm ratios at 0.74, 0.68 and 0.77, respectively. The highest NPQ values were measured in the combinations of BF/CA, CH/ZY and PW/SW at 3.06, 3.09 and 3.22, respectively, while the lowest values were in BF/PT, CH/CA and PW/639 at 1.50, 1.13 and 2.40, respectively. The combinations of BF/TO, CH/HJ and PW/MXT demonstrated the highest values of qP at 0.26, 0.31 and 0.31, respectively. Only the scion–rootstock interaction showed a significant effect on qP (Table 2). Among the 21 scion–rootstock combinations, the combinations of BF/TO, CH/HJ and PW/MXT had the highest qP values at 0.26, 0.31 and 0.31, respectively. Chislett and Powell grafted on Carrizo and X639 (CH/CA, PW/X639) had the lowest values at 0.11 and 0.14, respectively. Banfield grafted on MXT, Swingle and X639 all had the lowest qP at 0.17, significantly lower than on Trifoliate orange and Carrizo (Table 2).

### 2.3. Photosynthetic Pigments’ Content of the Citrus Scion–Rootstock Combinations

Among the scion, rootstock and scion–rootstock interaction, only rootstock had a significant effect on the content of chlorophylls, while none of the three ones had significant effect on the carotenoid content. As shown in Table 3, among the seven rootstocks, the effect of Ziyang xiangcheng on photosynthetic pigments was distinguishing. The highest contents of Chla, Chlb and Chlt were all observed in the combinations of Ziyang xiangcheng grafted with three scions (BF/ZY, CH/ZY, PW/ZY).

### 2.4. Rubisco Activity of the Citrus Scion–Rootstock Combinations

**Figure 1:**
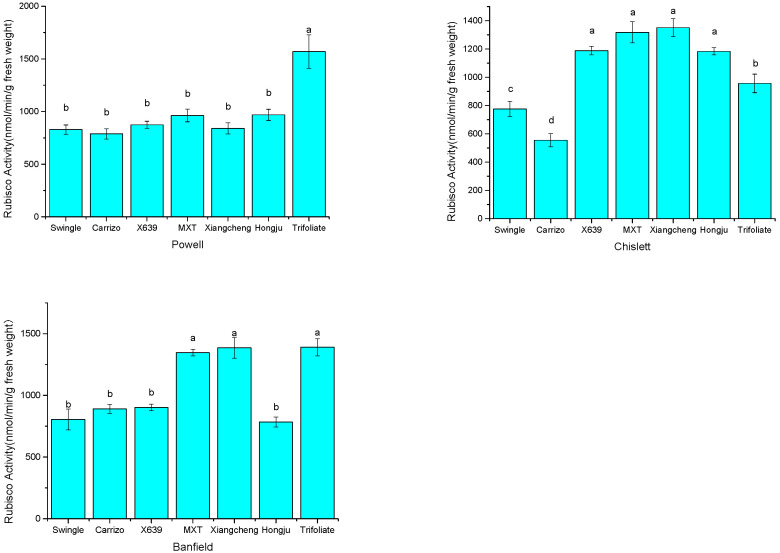
Rubisco activity of the citrus scion–rootstock combinations of the three scions. Note: different lower-cased letters indicate significant differences with Duncan’s multiple-range test at *p* < 0.05.

Only rootstock had a significant effect on the activity of Rubisco, but the effect was highly varied among the rootstocks. As shown in Figure 1, among the seven rootstocks, Trifoliate orange grafted with Banfield and Powell (BF/TO, PW/TO), and Ziyang xiangcheng with Chislett (CH/ZY) possessed the highest Rubisco activities at 1390.53, 1570.03 and 1350.34 μmol s^−1^ Kg^−1^ FW, respectively. Carrizo combined with Banfield and Chislett (BF/CA, CH/CA), and Hongju with Powell (PW/HJ) had the lowest activities at 788.00, 554.50 and 784.39 μmol s^−1^ Kg^−1^ FW, respectively.

## 3. Discussion

Scion, rootstock and their interaction can significantly affect the morphological and physiological performance of the plant [13]. The capacity of photosynthesis, which is essential for the synthesis of carbohydrates to support plant growth, is also influenced [37]. In this study, significant differences in the performance of photosynthesis were observed among the citrus scion–rootstock combinations. Liao et al. [38] reported that the citrus variety ‘Huangguogan’ (*C. reticulata*) grafted on Trifoliate orange, Hongju and Xiangcheng, which are the same three rootstocks included in our investigation, had different photosynthetic performances. In the present research, investigation of photosynthetic capacity was conducted in the 21 scion–rootstock combinations made by three scions and seven rootstocks, respectively, to illustrate the effect of scion, rootstock and scion–rootstock interaction on the performance of the photosynthesis and reveal their contributions to photosynthesis.

Fourteen photosynthesis indexes were investigated. Among the photosynthetic parameters, the content of chlorophylls (Chla, Chlb and Chlt) were significantly influenced by the rootstocks only. In the present research, we found that the content of photosynthetic pigments (Chla, Chlb, Chlt, and Car) of the combinations was the highest in the combinations using Ziyang xiangcheng as the rootstock (Table 3), indicating that the largest quantity of light quanta was probably absorbed in these combinations at the beginning of photosynthesis. Theoretically, approximately 30% light quanta absorbed by the photosystem could be transferred and conserved as biochemical energy [4,8]; the rest of light quanta is dissipated by other processes. The parameters such as NPQ and qP were important indicators for photoprotection against photoinhibition [39]. Non-photochemical quenching (NPQ), which is significantly influenced by both the scion and the scion–rootstock interaction (Table 2), indicating the dissipation of excess energy by fluorescence, and preventing deleterious photochemical reactions [8], differed significantly among the combinations. The NPQ value of the combinations using Ziyang xiangcheng as the rootstock was relatively high among the 21 scion–rootstock combinations (Table 2), indicating that large quantities of light quanta have been dissipated as fluorescence. qP, significantly influenced by the interaction of scion and rootstock (Table 2), indicates the light energy, which dissipates as heat or electron transport [9]. The qP value of the combinations using Trifoliate orange as the rootstock was also relatively high among the combinations (Table 2), which meant that a big portion of light quanta had been dissipated as heat. The interesting results were consistent with the phenomenon that citrus grafted on Ziyang xiangcheng was less severely burned by the sunlight than that grafted on Trifoliate in summer. To some extent, it could be ascribed to the good photoprotection with high NPQ values of citrus on the former rootstock, and the large quantity of heat dissipation with high qP of the latter one causing the second injury. It could probably provide a novel insight to evaluate the optimal rootstock for sunburn tolerance. The Fv/Fm ratio, one parameter of chloroplast fluorescence, is an important indicator to present the maximum potential ability of photosynthesis, the activity of the PSII components and an index to indicate whether a plant is under stress conditions [10]. For most of the plants under stress-free environment, Fv/Fm is close to 0.83 [39]. If the ratio is lower than this value, it indicates that the plant is under stressful conditions [10]. In our study, the Fv/Fm ratio of most combinations was close to 0.83, indicating that the plants of those scion–rootstock combinations were under good growing conditions. However, Fv/Fm ratios of some combinations such as Chislett and Powell on Carrizo rootstock (CH/CA, PW/CA) and Banfield on Trifoliate orang (BF/TO), were much lower than 0.83, and also significantly lower than those of the other combinations, indicating that the trees of the three combinations were probably under stressful conditions. Gonza’lez-Mas et al. [40] also indicated that the Fv/Fm value of ‘Navelina’ navel orange grafted on Carrizo was significantly lower than that on other rootstocks due to its least tolerance to calcareous condition, indicating that rootstocks could induce a contrasting photosynthesis response to change the tolerance to stresses [23] (Machado et al., 2013). However, the cause of this stress in our investigation was unknown. It is likely due to the fact that under natural field conditions, plants usually experience fluctuating light condition in timescales of seconds, minutes and hours [41].

Photosynthesis is the largest biological synthesis reaction on earth that converts the light energy into organic matter for plants [4]. In this research, we found that the rate of net CO_2_ assimilation of the grafted citrus plants was significantly influenced by the scion, the rootstock and their interaction among the citrus scion–rootstock combinations (Table 1). It is also influenced by many factors such as the concentration of intercellular CO_2_, the rate of transpiration and the stomatal conductance during the photosynthetic process. Compared with other combinations, the combinations of CH/CA and BF/TO possessed lower A_CO2_ and Ci, but higher G_H2O_, suggesting that their photosynthesis was probably affected by non-stomatal limitation. However, the combination PW/CA had relatively lower A_CO2_, and also low Ci and G_H2O_, showing that its photosynthesis was possibly influenced by stomatal limitation.

**Figure 2:**
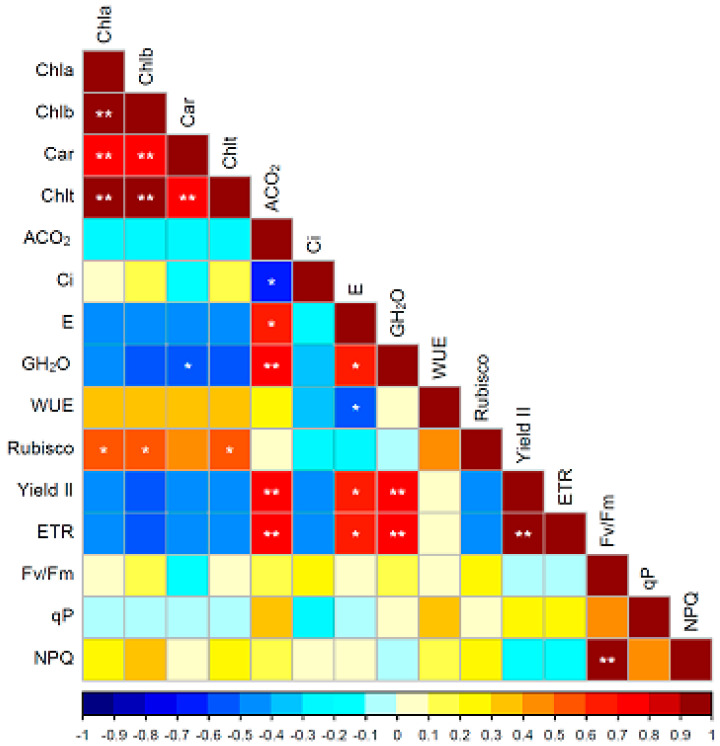
Correlation analysis of the parameters related to photosynthesis of the 21 citrus scion–rootstock combinations. Note: * and ** indicated the correlation with significant differences at *p* < 0.05 and *p* < 0.01, respectively. Chla, Chlb, Car and Chlt indicate chlorophyll a, b, carotenoids and total chlorophyll (Chlt), respectively. A_CO2_, Ci, E, G_H2O_, WUE, Yield II, ETR, Fv/Fm, qP and NPQ mean net CO_2_ assimilation rate, the concentration of intercellular CO_2,_ transpiration rate, stomatal conductance, water use efficiency, the quantum yield of the PSII, electron transport rate, the maximal quantum yield of PSII photochemistry, fluorescent quenching and non-photochemical quenching of fluorescence, respectively.

Electron transport is the most important process that converts the light energy into biochemical biomass [4]. The rate of electron transport is an essential parameter to reflect the internal characteristics of the photosynthetic system and the photosynthesis of plants under various conditions [42]. It was found that there was a significant and positive relationship between ETR and photosynthesis in maize (r = 0.8743 **) [9]. In this research, similar to the rate of net photosynthesis, ETR was also significantly influenced by the scion, the rootstock and their interaction (Table 2). The combinations which possessed the highest photosynthesis rate also had the highest electron transport rate (Table 1 and Table 2), suggesting that ETR was probably a predominate factor in determining the variation in photosynthesis among the scion–rootstock combinations. Coefficient analysis also indicated that net CO_2_ assimilation rate positively and significantly correlated to electron transport rate with r = 0.7791 ** (Figure 2), which further demonstrated their close relationship. Rubisco is the rate-limiting enzyme of the Calvin cycle and could determine the rate of net photosynthesis [4,43]. In the study of Liao et al. [38], the combination Huangguogan/Xiangcheng, which possessed a higher photosynthesis capacity, also had the highest Rubisco activity. However, this relationship was not observed in this study conducted in natural field conditions. In this investigation, the activity of Rubisco was significantly influenced by the rootstock only, not by the scion and the scion–rootstock interaction (Figure 1). The activity of Rubisco was the highest in the combinations of PW/TO, BF/TO and CH/ZY, but the highest rate of net photosynthesis (A_CO2_) was obtained in the combinations of PW/MXT, BF/SW and CH/SW. The inconsistencies between the activity of Rubisco and the rate of net photosynthesis in the scion–rootstock combinations could imply that there are probably some other factors that have influenced the photosynthesis. Heat and fluorescence dissipation indicated with qP and NPQ, which were significantly influenced by scion–rootstock interaction in Table 2, could illustrate these speculations. However, how they were influenced by the scion–rootstock interaction is rarely known. The effect of scion–rootstock interaction on the performances of the scion–rootstock combination was likely through the regulation of phytohormones, gene expression, long-distance transport of mRNAs, mediating effects of sRNAs, DNA methylation [13] or DNA demethylation [44]; however, the detailed information on how it is regulated needs to be further uncovered. Interestingly, we found that those combinations using Trifoliate orange as rootstock with high Rubisco activity combinations had the highest total soluble solid (TSS) content and fruit yield efficiency in our previous study [17]. This might imply that Rubisco, the most important enzyme in the Calvin cycle, is probably a predominate factor influencing the fruit quality and yield efficiency through regulating the carbohydrate accumulation.

## 4. Materials and Methods

### 4.1. Plant Materials

Three scion cultivars of late-ripening navel orange [*Citrus sinensis* cv. Powell (PW), Chislett (CH) and Banfield (BF)] grafted on seven citrus rootstocks, including Swingle citrumelo (SW, *C*. *paradisi* × *Poncirus trifoliata*), Carrizo citrange (CA, *C*. *sinensis* × *P*. *trifoliata*), X639 (*C*. *reticulata* × *P*. *trifoliata*), MXT (*C*. *sinensis* × *P*. *trifoliata*), Hongju (HJ, *C*. *reticulata*), Ziyang xiangcheng (ZY, *C*. *junos*) and Trifoliate orange (TO, *P*. *trifoliata*), were used as plant materials in this study. The 13-year-old experimental plants of the 21 scion–rootstock combinations, which were in good status and stable balance between the vegetative and reproductive growth, were grown in the same orchard in Fengjie County, Chongqing, Southwest China. It is located beside the Yangtse River with an average annual temperature of 17.7 °C, 1470.3 h of sunlight and 918.5 mm of precipitation. The altitude of the orchard is 342 m and the pH of the soil is 5.8. The planting density is 3.0 m × 4.0 m. Mature and healthy leaves from the third to sixth nodes of spring shoots in the out-layer of tree canopy were chosen for determining the parameter, three were sampled for photosynthesis and chlorophyll fluorescence measurement in field, six were sampled to the laboratory for the content of pigment detection, and the activity of Rubisco analysis. Three healthy plants of each scion–rootstock combination were randomly selected as three biological repeats, and three technique replicates were set for each biological repeat. The orchard was managed conventionally without irrigation.

### 4.2. Measurement of Leaf Photosynthesis and Chlorophyll Fluorescence

Measurement of photosynthesis was carried out at 8:30–11:00 a.m. on sunny days in summer. Photosynthetic gas exchange was measured using a GFS-3000 portable gas exchange system with a standard cuvette and a 3056-FL PAM-Fluorometer (Walz, Efferich, Germany). The reference CO_2_ concentration was kept at 400 mg·L^−1^. The measurement was carried out with temperature and relative humidity setting at 27 °C and 65%, respectively. Net CO_2_ assimilation rate (A_CO2_), stomatal conductance (G_H2O_), intercellular CO_2_ concentration (Ci) and transpiration rate (E) were measured with GFS-3000 portable gas-exchange system (Heinz Walz GmbH, Effeltrich, Germany) with 1000 µmol m^−2^ s^−1^ PAR (photosynthetic active radiation). The data was directly exported with GFS-Win software after the measurement stabilized.

Chlorophyll fluorescence was measured with photosynthetic gas exchange system at midnight under fully dark-adapted conditions. Energy partitioning parameters, presented as equations below, were calculated according to the method of Hendrickson et al. [45]. Effective quantum yield of PSII (Yield II) of a light-adapted sample reflecting the proportion of light absorbed by PSII was determined according to the following expression Yield II = (Fm′ − F)/Fm′, where Fm′ is the light-adapted maximum fluorescence and F is steady-state fluorescence. Electron transport rate (ETR) was calculated as ETR =PAR × Yield II × 0.84 × 0.5 [46]. The leaves’ initial parameters of PSII fluorescence—dark fluorescence yield (Fo) and the maximum fluorescence (Fm) were measured after dark adaptation. Effective quantum yields of PSII and NPQ of fluorescence were measured when the leaf acclimated full light to 1000 μmol photon m^−2^ s^−1^ actinic light. Maximum quantum yield of photosystem PSII was calculated as Fv/Fm = (Fm − Fo)/Fm, according to Genty et al. [46].

- Non-photochemical quenching of fluorescence (NPQ) and fluorescent quenching (qP) were calculated using the following equations [47,48,49]:
  NPQ = (Fm − Fm’)/Fm’ = Fm/Fm’ − 1
- qP = 1 − (F − Fo’calc)/(Fm’ − Fo’calc)

### 4.3. Photosynthetic Pigment Determination

One hundred milligrams of fresh leaves of each sample were collected and fully ground, then the photosynthetic pigments of leaves were extracted with 95% (*v*/*v*) ethanol. The contents of chlorophyll a (Chla), b (Chlb), total chlorophyll (Chlt) and carotenoids (Car) of the extracts were determined at maximum absorption peaks of 665 nm, 649 nm and 470 nm, respectively, according to the method described by Li [50]. The content of each pigment was calculated as follows:

Chla (mg/L) = 13.95A_665_ − 6.88A_649_

Chlb (mg/L) = 24.96A_649_ − 7.32A_665_

Chlt (mg/L) = Chla + Chlb

Car (mg/L) = (1000A_470_ − 2.05Ca − 114.8Cb)/245

### 4.4. Determination of Rubisco Activity

Activity of the important photosynthetic enzyme Rubisco was detected according to the manual of the Kit (Suzhou Grace Biotechnology Co. Ltd., China). One hundred milligram fresh leaves of each sample were weighed and fully pulverized. Then, 1 mL extracting solution was added, mixed and then centrifuged at 4 °C, 12,000 rpm for 10 min. The supernatant was transferred to a fresh tube for enzyme activity determination. The activity of the enzyme was detected based on an enzymatic method coupled to NADH oxidation of the extract before and after activation with CO_2_ and MgCl_2_ by spectrophotometer at 340 nm. The activation state was calculated as the ratio of the initial activity to the final activity (i.e., fully activated state) [51]. The activity of the enzyme was calculated as activity (µmol s^−1^ Kg^−1^ FW) = 10.72 × ΔA/0.1, ΔA = A1 − A2. FW indicated the weight of fresh leaf samples.

### 4.5. Statistical Analysis

The significant difference among the scion–rootstock combinations was determined with Duncan’s multiple-range test at *p* < 0.05. The significant impacts of scion and rootstock were analyzed with two-way ANOVA at *p* < 0.0001, as described by Tietel et al. [52]. Pearson correlation analysis was conducted with rcorr in Package Hmisc, and the correlation matrix was visualized with corrplot in Package corrplot. All the analyses in this study were conducted using R program (https://www.R-project.org/) accessed on 1 August 2024.

## 5. Conclusions

Scion, rootstock and their interaction had a significant effect on the processes of photosynthesis. Rootstock is the predominant factor that significantly affected the content of photosynthetic pigments. The rootstock and the scion–rootstock interaction had more effect on both the fluorescence and the photosynthesis of these scion–rootstock combinations with lower p values than the scion. Although many factors could influence the photosynthesis, the rate of electron transport influenced by all the scion, the rootstock and their interaction was the most essential factor that determined the final rate of photosynthesis, demonstrated by the correlation analysis in the citrus scion–rootstock combinations. In essence, rootstock and the scion–rootstock interaction had more effect on citrus photosynthesis via the effect on the rate of electron transport. This research is the first to reveal the different effects of the scion, the rootstock and their interaction on the photosynthesis of citrus scion–rootstock combinations, which is very valuable in enhancing our understanding on the different performances of citrus scion–rootstock combinations. It also aids to provide some novel insights on selecting the optimal scion–rootstock combinations in citriculture.

## Figures and Tables

**Table 1:** Photosynthetic parameters of the citrus scion–rootstock combinations.

Scions	Rootstocks	A_CO2_ (µmol m^−2^ s^−1^)	Ci (μmol mol^−1^)	E (mmol m^−2^ s^−1^)	G_H2O_ (mmol m^−2^ s^−1^)
BF	CA	7.82 ± 0.24 d	349.04 ± 3.00 a	2.43 ± 0.13 c	132.56 ± 0.77 bc
	HJ	6.67 ± 0.03 e	325.48 ± 2.80 bc	1.74 ± 0.03 e	96.73 ± 2.68 f
	MXT	8.93 ± 0.11 c	335.68 ± 3.76 b	2.84 ± 0.03 b	122.45 ± 1.89 de
	TO	9.75 ± 0.19 b	310.34 ± 4.86 d	2.74 ± 0.07 b	136.43 ± 3.79 b
	SW	11.22 ± 0.23 a	327.50 ± 3.17 bc	4.30 ± 0.08 a	163.92 ± 3.82 a
	X639	7.14 ± 0.25 e	353.97 ± 3.54 a	2.18 ± 0.03 d	117.69 ± 2.31 e
	ZY	8.82 ± 0.12 c	322.61 ± 4.20 c	2.21 ± 0.02 d	127.63 ± 2.66 cd
CH	CA	7.32 ± 0.16 d	357.05 ± 0.76 a	2.85 ± 0.10 b	110.82 ± 3.05 cd
	HJ	10.19 ± 0.21 a	304.88 ± 1.63 d	2.54 ± 0.08 c	120.24 ± 3.18 c
	MXT	8.34 ± 0.22 c	306.62 ± 2.53 d	1.68 ± 0.03 e	113.00 ± 1.23 cd
	TO	5.58 ± 0.25 e	388.59 ± 0.24 a	2.32 ± 0.08 cd	94.40 ± 6.34 d
	SW	10.77 ± 0.18 a	307.75 ± 3.92 d	2.38 ± 0.01 cd	159.31 ± 6.34 a
	X639	9.47 ± 0.09 b	322.22 ± 5.71 c	3.16 ± 0.11 a	146.67 ± 4.65 b
	ZY	8.47 ± 0.25 c	349.32 ± 2.35 b	2.28 ± 0.09 d	90.34 ± 7.51 d
PW	CA	7.62 ± 0.08 e	304.63 ± 3.21 d	3.00 ± 0.02 c	103.82 ± 1.63 c
	HJ	7.97 ± 0.04 d	344.45 ± 1.67 b	2.43 ± 0.05 d	138.04 ± 3.09 b
	MXT	12.27 ± 0.14 a	329.06 ± 5.87 c	3.91 ± 0.09 a	139.23 ± 5.02 b
	TO	10.12 ± 0.17 c	329.39 ± 0.98 c	3.27 ± 0.08 b	153.97 ± 1.88 a
	SW	11.69 ± 0.17 b	312.94 ± 2.23 d	3.36 ± 0.05 b	139.73 ± 1.44 b
	X639	8.11 ± 0.03 d	355.35 ± 1.84 a	2.38 ± 0.03 d	134.03 ± 2.65 b
	ZY	7.07 ± 0.04 f	336.30 ± 4.22 bc	1.78 ± 0.04 d	97.38 ± 3.60 c
*p*	Scion	1.29 × 10^−9^ ***	0.7012	4.19 × 10^−15^ ***	6.05 × 10^−9^ ***
	Rootstock	1.74 × 10^−27^ ***	0.00015 **	1.00 × 10^−25^ ***	1.68 × 10^−23^ ***
	Interaction	8.91 × 10^−26^ ***	8.47 × 10^−8^ ***	3.25 × 10^−28^ ***	1.10 × 10^−21^ ***

Note: BF, CH and PW indicate the three scion cultivars of ‘Banfield’, ‘Chislett’ and ‘Powell’, respectively. CA, HJ, MXT, TO, SW, X639 and ZY are the seven rootstock varieties of ‘Carrizo’, ‘Hongju’, ‘MXT’, ‘Trifoliate orange’, ‘Swingle’, ‘X639’ and ‘Ziyang xiangcheng’, respectively. Data are means ± standard error obtained from three biological repeats with three technical replicates (n = 9). Different lower-cased letters indicate significant differences with Duncan’s multiple-range test at *p* < 0.05. ** and *** indicate the significance at *p* < 0.001 and *p* < 0.0001 with two-way ANOVA, respectively.

**Table 2:** Chlorophyll fluorescence of the citrus scion–rootstock combinations.

Scions	Rootstocks	Yield II	ETR (µmol Electrons m^−2^ s^−1^)	Fv/Fm	qP	NPQ
BF	CA	0.28 ± 0.03 bc	116.31 ± 2.23 b	0.83 ± 0.00 a	0.22 ± 0.00 a	3.06 ± 0.02 a
	HJ	0.20 ± 0.02 b	81.88 ± 1.20 d	0.80 ± 0.01 ab	0.21 ± 0.05 a	2.62 ± 0.09 a
	MXT	0.31 ± 0.03 a	125.69 ± 3.26 a	0.84 ± 0.00 a	0.17 ± 0.04 b	2.90 ± 0.09 a
	TO	0.28 ± 0.03 ab	114.71 ± 1.68 b	0.74 ± 0.04 b	0.26 ± 0.17 a	1.50 ± 0.56 b
	SW	0.32 ± 0.03 a	132.24 ± 4.15 a	0.83 ± 0.00 a	0.17 ± 0.04 b	2.70 ± 0.07 a
	X639	0.28 ± 0.04 ab	112.33 ± 3.53 b	0.82 ± 0.01 a	0.17 ± 0.01 b	2.29 ± 0.21 ab
	ZY	0.23 ± 0.02 ab	94.42 ± 0.94 c	0.80 ± 0.01 ab	0.19 ± 0.02 ab	2.71 ± 0.41 a
CH	CA	0.27 ± 0.03 bc	110.82 ± 2.14 d	0.68 ± 0.02 b	0.11 ± 0.00 b	1.13 ± 0.22 d
	HJ	0.29 ± 0.03 abc	120.24 ± 2.83 d	0.81 ± 0.01 a	0.31 ± 0.10 a	2.98 ± 0.18 ab
	MXT	0.27 ± 0.02 bc	113.00 ± 3.20 d	0.79 ± 0.02 a	0.17 ± 0.02 ab	2.03 ± 0.07 d
	TO	0.23 ± 0.02 c	94.09 ± 0.64 e	0.82 ± 0.01 a	0.22 ± 0.03 ab	2.57 ± 0.04 abc
	SW	0.38 ± 0.04 a	158.39 ± 2.08 a	0.77 ± 0.02 ab	0.20 ± 0.03 ab	1.89 ± 0.14 bcd
	X639	0.36 ± 0.04 ab	146.67 ± 2.22 b	0.80 ± 0.03 a	0.29 ± 0.01 a	2.68 ± 0.58 abc
	ZY	0.22 ± 0.02 c	90.22 ± 0.26 e	0.81 ± 0.01 a	0.24 ± 0.02 ab	3.09 ± 0.39 a
PW	CA	0.28 ± 0.02 bc	116.75 ± 3.60 c	0.77 ± 0.04 b	0.21 ± 0.02 ab	2.56 ± 0.30 ab
	HJ	0.24 ± 0.04 c	100.31 ± 1.28 d	0.82 ± 0.01 a	0.24 ± 0.03 ab	2.84 ± 0.23 ab
	MXT	0.36 ± 0.04 ab	145.48 ± 0.97 b	0.82 ± 0.00 a	0.31 ± 0.02 a	3.13 ± 0.05 a
	TO	0.27 ± 0.03 bc	110.92 ± 2.15 c	0.82 ± 0.00 a	0.22 ± 0.03 ab	3.08 ± 0.04 a
	SW	0.38 ± 0.04 a	154.59 ± 3.33 a	0.82 ± 0.00 a	0.30 ± 0.07 a	3.22 ± 0.10 a
	X639	0.22 ± 0.02 c	90.85 ± 1.03 e	0.83 ± 0.01 a	0.14 ± 0.00 b	2.40 ± 0.07 b
	ZY	0.20 ± 0.02 c	83.00 ± 0.57 f	0.81 ± 0.01 a	0.17 ± 0.02 b	2.77 ± 0.28 ab
*p*	Scion	0.5235	0.00275 *	0.0068 *	0.2838	0.0033 *
	Rootstock	7.68 × 10^−6^ ***	1.95 × 10^−26^ ***	0.0114	0.2214	0.0606
	Interaction	0.0169	7.85 × 10^−18^ ***	0.0014 *	0.0071 *	0.0004 **

Note: BF, CH and PW indicate the three scion cultivars of ‘Banfield’, ‘Chislett’ and ‘Powell’, respectively. CA, HJ, MXT, TO, SW, X639 and ZY are the seven rootstock varieties of ‘Carrizo’, ‘Hongju’, ‘MXT’, ‘Trifoliate orange’, ‘Swingle’, ‘X639’ and ‘Ziyang xiangcheng’, respectively. Data are means ± standard error obtained from three biological repeats with three technical replicates (n = 9). Different lower-cased letters indicate significant differences with Duncan’s multiple-range test at *p* < 0.05. *, ** and *** indicate the significance at *p* <0.01, *p* < 0.001 and *p* < 0.0001 with two-way ANOVA, respectively.

**Table 3:** Content of photosynthetic pigments of the citrus scion–rootstock combinations.

Scions	Rootstocks	Chla (μmol s^−1^ Kg^−1^ FW)	Chlb (μmol s^−1^ Kg^−1^ FW)	Car (μmol s^−1^ Kg^−1^ FW)	Chlt (μmol s^−1^ Kg^−1^ FW)
BF	CA	12.69 ± 0.18 a	5.52 ± 0.16 ab	2.43 ± 0.25 ab	18.21 ± 0.31 a
	HJ	12.43 ± 0.50 a	5.41 ± 0.28 ab	2.69 ± 0.01 a	17.84 ± 0.75 ab
	MXT	12.32 ± 0.15 a	5.44 ± 0.18 ab	2.52 ± 0.09 ab	17.75 ± 0.33 ab
	TO	12.05 ± 0.38 b	5.12 ± 0.15 b	2.49 ± 0.13 ab	17.17 ± 0.50 b
	SW	11.10 ± 0.12 c	4.64 ± 0.14 c	2.25 ± 0.03 b	15.74 ± 0.24 c
	X639	12.91 ± 0.08 a	5.53 ± 0.02 ab	2.56 ± 0.09 ab	18.44 ± 0.09 a
	ZY	13.68 ± 0.23 a	6.15 ± 0.20 a	2.42 ± 0.05 ab	19.83 ± 0.40 a
CH	CA	12.06 ± 0.03 c	5.18 ± 0.08 b	2.33 ± 0.12 bc	17.24 ± 0.10 c
	HJ	11.19 ± 0.50 c	4.62 ± 0.19 c	2.31 ± 0.11 bc	15.81 ± 0.69 c
	MXT	11.15 ± 0.36 c	4.98.0 ± 0.20 bc	2.09 ± 0.05 c	16.13 ± 0.55 c
	TO	11.43 ± 0.08 c	4.74 ± 0.06 c	2.11 ± 0.16 c	16.17 ± 0.15 c
	SW	11.21 ± 0.53 c	4.53 ± 0.22 c	2.24 ± 0.08 bc	15.74 ± 1.18 c
	X639	12.72 ± 0.81 b	5.42 ± 0.37 b	2.42 ± 0.21 ab	18.14 ± 1.15 b
	ZY	14.33 ± 0.34 a	6.64 ± 0.25 a	2.81 ± 0.06 a	20.97 ± 0.69 a
PW	CA	11.83 ± 0.44 cd	5.10 ± 0.28 cd	2.44 ± 0.19 ab	16.93 ± 0.68 de
	HJ	11.52 ± 0.21 e	5.22 ± 0.20 cd	2.21 ± 0.08 b	16.74 ± 0.41 e
	MXT	11.79 ± 0.23 de	5.24 ± 0.03 cd	2.24 ± 0.05 b	17.03 ± 0.25 cd
	TO	11.37 ± 0.33 e	4.82 ± 0.17 d	2.19 ± 0.13 b	16.19 ± 0.48 e
	SW	12.89 ± 0.66 bc	5.62 ± 0.35 bc	2.34 ± 0.15 ab	18.51 ± 1.01 bc
	X639	13.14 ± 0.15 ab	5.80 ± 0.07 b	2.52 ± 0.15 ab	18.94 ± 0.19 b
	ZY	13.98 ± 0.49 a	6.41 ± 0.13 a	2.63 ± 0.21 a	20.39 ± 0.61 a
*p*	Scion	0.3020	0.1502	0.0213	0.2589
	Rootstock	7.21 × 10^−6^ ***	5.04 × 10^−8^ ***	0.0157	1.32 × 10^−6^ ***
	Interaction	0.5623	0.3350	0.0719	0.4847

Note: BF, CH and PW indicate the three scion cultivars of ‘Banfield’, ‘Chislett’ and ‘Powell’, respectively. CA, HJ, MXT, TO, SW, X639 and ZY are the seven rootstock varieties of ‘Carrizo’, ‘Hongju’, ‘MXT’, ‘Trifoliate orange’, ‘Swingle’, ‘X639’ and ‘Ziyang xiangcheng’, respectively. Data are means ± standard error obtained from three biological repeats with three technical replicates (n = 9). Different lower-cased letters indicate significant differences with Duncan’s multiple-range test at *p* < 0.05. *** indicates the significance at *p* < 0.0001 with two-way ANOVA.

## Data Availability

The original contributions presented in this study are included in the article. Further inquiries can be directed to the corresponding author.
